# Contrast medium‐induced transient severe leukopenia

**DOI:** 10.1002/ams2.319

**Published:** 2017-10-25

**Authors:** Takashi Hongo, Satoshi Nozaki, Midori Tsuchiya, Mototaka Inaba, Kenji Takahashi, Toshifumi Fujiwara

**Affiliations:** ^1^ Emergency Department Okayama Saiseikai General Hospital Okayama Japan

**Keywords:** Allergy, computed tomography, contrast media, drug‐induced leukopenia, ER

## Abstract

**Case:**

Contrast medium‐induced transient leukopenia is very rare. Here, we report a case of a 73‐year‐old man diagnosed with contrast media‐induced transient leukopenia. The patient underwent abdominal contrast‐enhanced computed tomography, where he was given non‐ionic iodinated contrast medium i.v. His medical history included an allergic reaction to a different contrast medium. One hour later, the patient was admitted to the emergency department complaining of chest discomfort. He had leukopenia and a fever (temperature of 38.9°C). Complete blood count showed a white blood cell count of 930/μL and an absolute neutrophilic count of 232/μL.

**Outcome:**

The patient was given i.v. antibiotics and 5 mg chlorpheniramine maleic acid, 20 mg famotidine, and 125 mg methylprednisolone. The patient's white blood cell count recovered the next day, and he was discharged after 2 days of hospitalization.

**Conclusion:**

We diagnosed the patient with contrast media‐induced transient leukopenia, which is a rare phenomenon.

## Introduction

Contrast media is frequently used in radiology. It is an essential tool for medical diagnosis and interventional radiology. Few studies have implicated contrast media use in the development of leukopenia.^1^, ^2^ Agranulocytosis is a life‐threating condition that occurs due to decreased absolute neutrophil counts of <500/μL.^3^ Here, we report a case of a 73‐year‐old man diagnosed with contrast media‐induced transient leukopenia.

## Case

**Figure 1:**
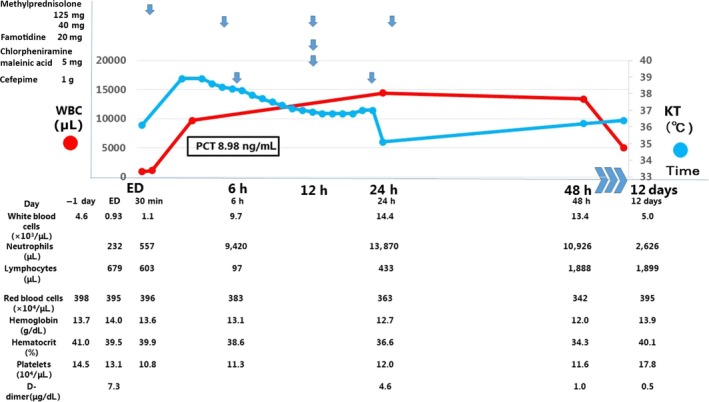
Clinical course of a 73‐year‐old man with contrast media‐induced transient leukopenia. The figure depicts changes in the patient's white blood cell count (WBC), absolute neutrophil count, and body temperature over time. WBC count was significantly decreased. The patient had a fever temperature of 38.9°C at 4 h after admission to the emergency department (ED). His WBC count gradually improved. The patient was discharged 2 days later. KT, körpertemperatur; PCT, procalcitonin.

At 10:08 am, a 73‐year‐old‐man underwent an abdominal contrast‐enhanced computed tomography (CT) scan using non‐ionic iodinated contrast medium, as a follow‐up procedure for chronic pancreatitis. The procedure was completed uneventfully and the CT showed no significant findings. One hour after the procedure, he was admitted to the emergency department for chest discomfort. The patient had a medical history of chronic pancreatitis and allergy to a different contrast medium with symptoms of only nausea that occurred 6 years ago. Thus, we changed the contrast medium and used them 10 times. No symptoms occurred before the present case. The patient's initial findings were: height, 173 cm; weight, 77.0 kg; blood pressure, 83/52 mmHg; heart rate, 40 b.p.m.; temperature, 36.1°C; respiratory rate, 18/min; SpO_2_, 98% with oxygen delivered through a face mask (5 L/min); and a Glasgow Coma Scale score of E3V5M6. A physical examination revealed normal bilateral air entry without any wheezing or rhonchi. The results of his cardiovascular and neurological examinations were normal. His abdomen on examination appeared normal and did not show any skin eruptions. The clinical course of the patient is shown in Fig. 1.

**Figure 2:**
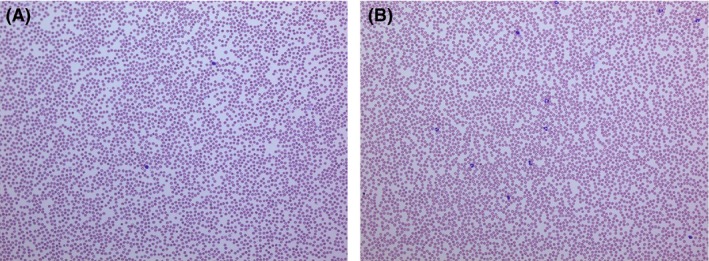
Hematoxylin–eosin staining (×10) of peripheral blood cells from a 73‐year‐old man with contrast media‐induced transient leukopenia. A, Neutrophils are not present in the peripheral blood at the time of admission to the emergency department. B, Normal neutrophils are present in the peripheral blood 12 days later.

The laboratory findings are shown in Table 1. Both the electrocardiogram and chest X‐ray did not reveal any significant findings. The patient's total white blood cell, neutrophil, and lymphocyte counts were 930, 232, and 678/μL, respectively (Fig. 2A). His hemoglobin levels and platelet counts were normal. Initially, anaphylaxis was suspected due to his medical history and cardiovascular symptoms. Thus, the patient was given 5 mg chlorpheniramine malcinic acid, 20 mg famotidine, and 125 mg methylprednisolone, and admitted to the hospital for follow‐up.

**Table 1:** Laboratory testing of a 73‐year‐old man with contrast media‐induced transient leukopenia at admission to the emergency department (ED), 1 day before admission, and 12 days after admission

	−1 day	ED	+12 days
Peripheral blood			
Red blood cells, ×10^4^/μL	398	370	395
Hemoglobin, g/dL	13.7	14.0	13.9
Hematocrit, %	41.0	39.5	40.1
Platelets, ×10^4^/μL	14.5	13.1	17.8
White blood cells, /μL	4,690	930	5,050
Band, %	ND	1.0	52.9
Segmented, %	ND	24.0	1.8
Eosinophils, %	ND	1.0	1.8
Basophils, %	ND	0.0	0.6
Monocytes, %	ND	1.0	7.1
Lymphocytes, %	ND	73.0	37.6
Blood chemistry			
Total protein, g/dL	7.1	6.3	7.0
Albumin, g/dL	4.5	3.9	4.2
Urea nitrogen, mg/dL	9.2	10.5	11.2
Creatinine, mg/dL	0.83	0.85	0.89
Aspartate aminotransferase, IU/L	23	22	20
Alanine aminotransferase, IU/L	21	22	23
Total bilirubin, mg/dL	ND	1.1	1.4
Alkaline phosphatase, IU/L	ND	168	175
Lactic acid dehydrogenase, U/L	ND	163	155
Sodium, mEq/L	141	137	140
Potassium, mEq/L	3.7	3.9	4.2
Chloride, mEq/L	105	105	106
Glucose, mg/dL	126	165	140
Serological test			
C‐reactive protein test, mg/dL	ND	0.04	0.15
Coagulation			
Prothrombin time, s	ND	11.5	11.9
Activated partial thromboplastin time, s	ND	23.2	30.8
D‐dimer, μg/mL	ND	7.3	0.5
IL‐6, pg/mL	ND	2.9	ND
TNF‐α, pg/mL	ND	0.97	ND
Anti‐neutrophil antibodies	ND	Negative	ND

IL‐6, interleukin‐6; TNF‐α, tumor necrosis factor‐α; ND, not done laboratory finding.

Shortly after admission, the patient complained of chills and had a temperature of 37.6°C. His total white blood cell count was 1,160/μL and the neutrophil count was 557/μL. Approximately 2 h after admission the patient developed a fever and his body temperature was 38.9°C. We obtained samples of blood, sputum, and urine for culture. Subsequent whole‐body CT scans and a chest X‐rays did not reveal any infection foci. The patient was diagnosed with neutropenic fever and treated with cefepime. At 3:00 am the next day, his temperature returned to normal. In addition, his white blood cell count had fully recovered. Microbial culture results were negative for bacterial infection. Consequently, we diagnosed the patient with idiosyncratic transient leukopenia that was induced by the contrast medium. Interleukin‐6, tumor necrosis factor‐α (TNF‐α), and anti‐neutrophil antibody findings were unremarkable. The condition of the patient improved on day two after hospitalization and he was discharged (Fig. 2B). The patient has not experienced a recurrence in the 1 year since this event.

## Discussion

There are few reports of contrast medium‐induced acute leukopenia. We undertook a thorough search of published work using PubMed with the following search words: contrast‐related leukopenia; immunosuppression; and anaphylaxis‐related leukopenia. We identified a report by Kovoor and Morgan, describing a case of severe transient leukopenia following a hysterosalpingography after the use of an iodinated contrast medium.^2^ In this case, at 4 h after the hysterosalpingography, a full blood count revealed severe leukopenia. The white blood cell counts gradually improved at 3 h after admission and a blood culture was negative. The study suggests that the late reaction we observed may be a late allergic reaction.

Agranulocytosis is defined as an absolute neutrophil count of <500/μL.^3^ It is widely known that anticancer agents induce agranulocytosis.^4^ Idiosyncratic drug‐induced agranulocytosis (IDIAG) occurs at a rate of 3–15 cases per million inhabitants per year.^5^ It is a life‐threating disease because of sepsis, and its mortality rate is estimated as 5–20%.^6^ Most cases of IDIAG are due to the use of antiplatelets, antithyroids, clozapine, beta‐lactams, and vancomycin. Old age, sex (female), renal failure, and autoimmune disease are also risk factors for agranulocytosis.^7^ The diagnosis of IDIAG requires a detailed medication history and a high index of suspicion for possible offending medications. In addition, DIAG diagnosis requires a <500 cell/mm^3^ neutrophil count, >10 g/dL hemoglobin level, >100,000 cell/mm^3^ platelet count, a history of drug exposure, and no previous indication of a secondary cause of agranulocytosis.^3^ The patient in our case did not receive any medication other than contrast medium or have a history of neutropenia. His white blood cell count and differentiation were normal the day before the condition developed.

Although the pathophysiology of contrast media‐induced leukopenia is unknown, it may result from toxicity and altered immune functions.^8^ Few studies have investigated the effects of contrast medium on leukocyte count *in vitro*.^9^ One report suggested that iodinated contrast media induced apotosis^10^ through p38 mitogen‐activated protein kinase (MAPK). The p38 MAPK kinase is activated by a variety of cellular stresses including inflammatory cytokines, lipopolysaccharides, UV irradiation, and hyperosmolality.^10^ Laboratory findings in this case revealed that interleukin‐6 was 2.9 pg/mL. Our patient had an allergy history to a different contrast medium. We suspect that the patient had an immune mechanism for leukopenia due to a previous exposure to contrast medium; thus, he showed a rapid onset of transient leukopenia. Human leukocyte antigen genes (HLA) and other genes associated with an immune response were associated with an increased risk of leukopenia.^8^ Anti‐neutrophil antibodies are present in immune‐mediated, drug‐induced leukopenia. The immune response that results in leukopenia may involve neutrophil oxidation to a reactive metabolite or a neutrophil functional group that has the potential to be easily oxidized into a reactive metabolite.^8^ However, in the present case, we did not detect anti‐neutrophil antibodies.

Leukopenia also occurs in severe sepsis. Tumor necrosis factor‐α has an important role in sepsis; it affects white blood cells and neutrophil apoptosis. However, our laboratory findings revealed that the TNF‐α level was 0.97 pg/mL.

The occurrence of leukopenia after the administration of contrast media is rare. In our case, leukopenia appeared soon after administration of the contrast medium and recovered relatively early. Moreover, recent evidence suggests that the drugs that cause leukopenia can activate inflammasomes.

## Conclusion

This is a rare case of transient leukopenia that was caused by contrast medium. More reports of such rare cases are needed to confirm our findings.

## Disclosure

Approval of the research protocol: Yes, informed consent was obtained from the patient.

Informed consent: acceptable.

Registry and the registration no. of the study/trial: None.

Animal studies: None.

Conflict of interest: None.
